# Daily Subjective Nearness to Death and Well-Being Among Older Adults of Different Religious Affiliations

**DOI:** 10.1093/geroni/igaf122.1492

**Published:** 2025-12-31

**Authors:** Amit Shrira, Shevaun Neupert, Dwight Tse, Reyyan Can, Yuval Palgi

**Affiliations:** Bar-Ilan University, Ramat Gan, HaMerkaz, Israel; North Carolina State University, Raleigh, North Carolina, United States; University of Strathclyde, Glasgow, Scotland, United Kingdom; North Carolina State University, Raleigh, North Carolina, United States; University of Haifa, HaZafon, Israel

## Abstract

Subjective nearness to death (SNtD) refers to how close one perceives oneself to death. It is related to well-being indices and is even associated with actual life expectancy. While daily variations were demonstrated in different subjective views of aging, whether SNtD fluctuates and covaries with well-being in short-term time frames remains unclear. Moreover, although SNtD variations and concomitants may be affected by religious belief systems, such effects are still underexplored. We capitalized on data from the Subjective AGES (Aging within Global Everyday Ecological Studies) project examining daily changes in SNtD and their covariation with well-being indices: somatic symptoms, memory failures, and negative affect. We further assessed daily changes and covariations among older adults with various religious affiliations: Christians (from the UK and USA), Muslims (from Türkiye), and Jews (from Israel). SNtD showed significant daily fluctuations (within-person variance ranged from 20% to 35% across the different religious affiliations). It further correlated with daily well-being so that on days when participants felt closer to death than usual, they reported more somatic symptoms, more memory failures, and higher negative affect. In addition, significant moderation effects for religious affiliation showed that the daily covariation between SNtD and some well-being indices was weaker among Muslims but stronger among Jews. The findings indicate that SNtD exhibits significant daily variations, which coincide with older adults’ daily physical, cognitive, and emotional experiences. While SNtD was relevant to the well-being of older individuals with varying religious affiliations, religious belief systems, including beliefs about the afterlife, modify the SNtD-well-being relationship.

